
JOURNAL INFORMATION
==============================
NLM Title Abbreviation: ScientificWorldJournal
Journal ID: ScientificWorldJournal
NLM Journal ID: 101131163
Journal ID: TSWJ

The Scientific World Journal

ISSN: 2356-6140
EISSN: 1537-744X
Publisher: Wiley

ARTICLE INFORMATION
==============================
PMCID: PMC5956353
PMID: 15578125
DOI: 10.1100/tsw.2004.202
Article ID: 109707
Article version: 1
Subjects: Case Study

Extensive Urinary System Lymphoma Delineated by Coronal Computed Tomography

Ooi Jason
Elmes Martin R.
Lawrentschuk Nathan *
University of Melbourne, Department of Surgery and Department of Urology, Austin Hospital, Studley Road, Heidelberg, Victoria, 3084 and Department of Surgery, Western Hospital, Gordon Street, Footscray Vic, 3011, Australia
*Corresponding author.Academic Editor: Anthony Atala

Publication date: 2004
Electronic publication date: 2004 Nov 20
Volume: 4
First page: 1014
Last page: 1016
Received 2004 Jul 15; Revised 2004 Nov 7; Accepted 2004 Nov 11
Copyright: Copyright © 2004 Jason Ooi et al.
Copyright year: 2004
License: This is an open access article distributed under the Creative Commons Attribution License, which permits unrestricted use, distribution, and reproduction in any medium, provided the original work is properly cited.
License URL: https://creativecommons.org/licenses/by/3.0/

Keywords: lymphoma, kidney, ureter, computed tomography, ureteral obstruction

==============================
Case Study
*TheScientificWorldJOURNAL* (2004) 4, 1014–1016
ISSN 1537-744X; DOI 10.1100/tsw.2004.202

### Extensive Urinary System Lymphoma Delineated by Coronal Computed Tomography

Jason Ooi, Martin R. Elmes, and Nathan Lawrentschuk*
*University of Melbourne, Department of Surgery and Department of Urology, Austin Hospital, Studley Road, Heidelberg, Victoria, 3084 and Department of Surgery, Western Hospital, Gordon Street, Footscray Vic, 3011, Australia*

**E-mail**: nayjay@ozemail.com.au

*Received July 15, 2004; Revised November 7, 2004; Accepted November 11, 2004; Published November 20, 2004*

**KEY WORDS:** lymphoma, kidney, ureter, computed tomography, ureteral obstruction

**DOMAINS:** imaging, clinical medicine, surgery, medical care

### CASE REPORT

A 44-year-old male petrochemical worker presented with 3 weeks of right loin pain and lethargy. Excessive consumption of nonsteroidal anti-inflammatory medications for analgesia resulted in upper gastrointestinal bleeding, renal failure, and anaemia. After medical stabilisation, abdominal computed tomography (CT) demonstrated a soft tissue mass arising from the left kidney, infiltrating the ureter with diffuse bladder wall thickening (Figs. 1 and 2). The obstructed left collecting system was relieved initially with a percutaneous nephrostomy that was later substituted by an antegrade ureteric stent. Rigid cystoscopy revealed a featureless and intact bladder urothelium, but biopsy demonstrated diffuse large B cell lymphoma, later confirmed on bone marrow aspiration. Chemotherapy was instituted (CHOP: Cyclophosphamide, Doxorubicin, Oncovine, Prednisolone) and repeat CT after two cycles of chemotherapy and ureteric stent removal demonstrated complete resolution of disease (Fig. 3).

### DISCUSSION

Urologists are rarely consulted about lymphoma due to the paucity of symptoms[1] despite the incidence of genitourinary tract involvement by advanced lymphoma being up to 52% in autopsy series[2]. This case highlights an unusual symptomatic presentation from extensive renal, ureteric, and bladder involvement and the value of CT with coronal reformatting to delineate the extent of disease. Diagnoses other than urothelial tumours need to be considered when confronted with such extensive disease and profound ureteric compression[3]. Finally, with appropriate histological diagnosis and treatment, lymphoma may undergo a dramatic resolution, as demonstrated in this case.

*Corresponding author.
©2004 with author.

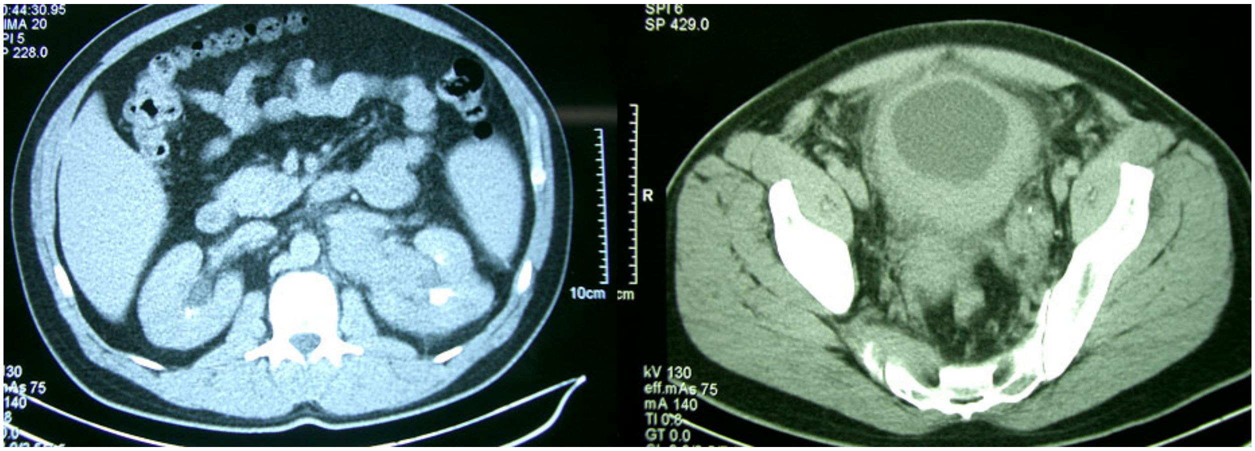

**FIGURE 1.** Axial computed tomography images demonstrating widespread involvement of the urinary tract by lymphoma causing obstruction (left) with widespread bladder wall involvement (right).

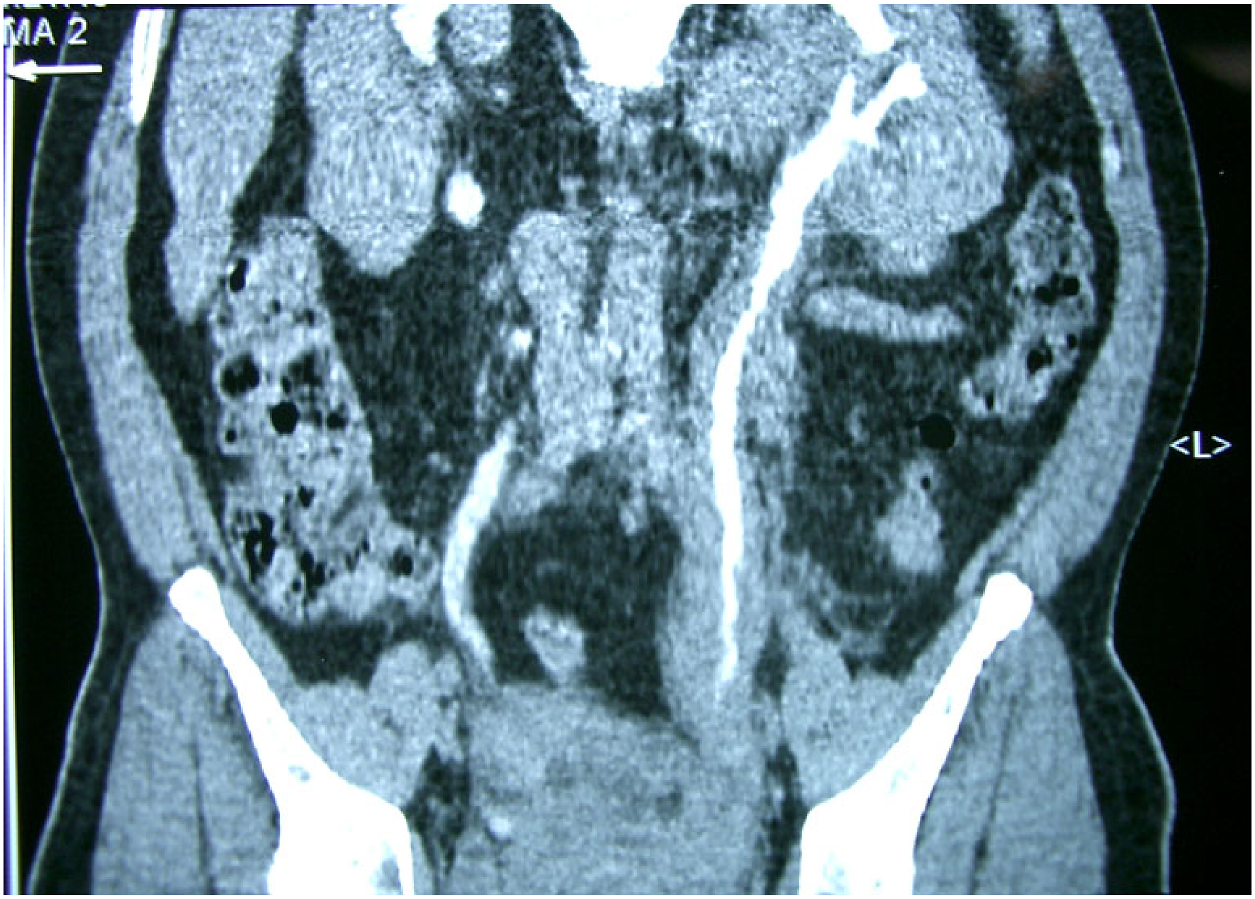

**FIGURE 2.** Coronal reformatting of the computed tomography images in Fig. 1 highlighting the extensive involvement of bladder and in particular the left ureter and kidney.

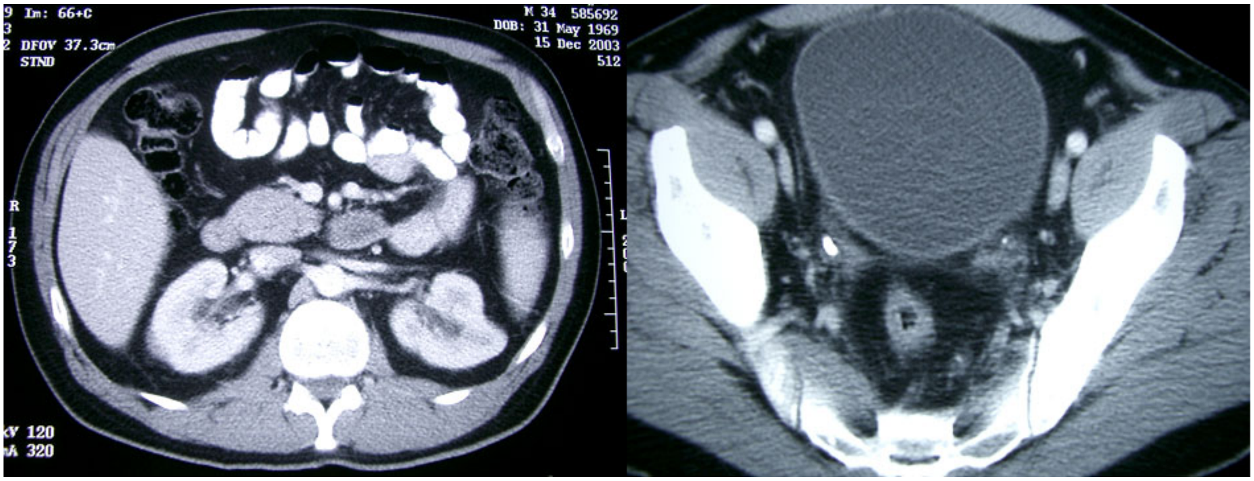

**FIGURE 3.** Axial computed tomography images demonstrating resolution of the previous extensive urinary tract lymphoma and its associated obstruction (left) with the bladder wall now normal (right).

### REFERENCES

1. Salem, Y.H. and Miller, H.C. (1994) Lymphoma of genitourinary tract. *J. Urol.* **151**, 1162–1170.
2. Lalli, A.F. (1969) Lymphoma and the urinary tract. *Radiology* **93**, 1051–1054.
3. Charnsangavej, C. (1990) Lymphoma of the genitourinary tract. *Radiol. Clin. North Am.* **28**, 865–877.

**This article should be referenced as follows:**

Ooi, J., Elmes, M.R., and Lawrentschuk, N. (2004) Extensive urinary system lymphoma delineated by coronal computed tomography. *TheScientificWorldJOURNAL* **4**, 1014–1016.

**Handling Editor:**

Anthony Atala, Principal Editor for *Urology* and *Tissue Engineering*— domains of *TheScientificWorldJOURNAL*.

### BIOSKETCHES

**Jason Ooi, MB, BS,** Urology Registrar, University of Melbourne, Department of Surgery and Department of Urology , Austin Hospital, Studley Road, Heidelberg, Victoria, 3084. Australia.

**Martin R. Elmes, MB, BS,** Urology Resident, Department of Urology, Western Hospital, 148 Gordon Street, Footscray Vic, 3011, Australia.

**Nathan Lawrentschuk MB, BS,** Urology Research Fellow, University of Melbourne, Department of Surgery and Department of Urology, Austin Hospital, Studley Road, Heidelberg, Victoria, 3084. Australia.
